# Nitrergic relaxation of gastrointestinal smooth muscle: role of interstitial cells of Cajal and smooth muscle cells

**DOI:** 10.1186/1471-2210-11-S1-P44

**Published:** 2011-08-01

**Authors:** Barbara Lies, Dieter Groneberg, Dieter Saur, Peter König, Andreas Friebe

**Affiliations:** 1 Physiologisches Institut, Universität Würzburg, Germany; 2 Medizinische Klinik (Gastroenterologie), Klinikum rechts der Isar, München, Germany; 3 Institut für Anatomie, Universität Lübeck, Germany

## Background

The signaling molecule nitric oxide (NO) is known to activate the enzyme NO-sensitive guanylyl cyclase (NO-GC). By generation of the intracellular second messenger cGMP NO-GC regulates many physiological processes. In the gastrointestinal (GI) tract nitrergic neurons are part of the enteric nervous system which regulates GI motility. In addition, interstitial cells of Cajal (ICC) are thought to be involved in nitrergic relaxation. In this study, we intended to clarify the role of NO-GC regarding GI motility in mice by using cell-specific knockout strains.

## Methods

We have generated mouse lines that lack NO-GC ubiquitously (total GCKO), specifically in smooth muscle (SM-GCKO) or in ICC (ICC-GCKO) as well as in both smooth muscle cells (SMC) and ICC (dbl-GCKO). By means of isometric force studies the effects of NO were investigated in these mice. Total gut transit time was measured to monitor the consequences of NO-GC deletion on gut motility in vivo. Furthermore, immunohistological staining was conducted to ascertain the distribution of NO-GC in the different GI cell types.

## Results

NO-dependent relaxation of GI smooth muscle was abolished in total GCKO mice. In comparison to WT whole gut transit time was increased. Surprisingly, in SM-GCKO, NO-dependent relaxation was hardly affected and total gut transit time showed no difference to WT controls. Similarly, a WT-like phenotype was observed in ICC-specific knockout mice (ICC-GCKO). Only in double knockouts we observed a phenotype similar to that seen in total GCKO mice including lack of nitrergic relaxation and increased gut transit time. Immunohistochemistry revealed NO-GC expression in SMC and ICC as well as in a third cell type; this cell type could be characterized as fibroblast-like cells (FLC).

## Conclusion

In conclusion, the NO receptor guanylyl cyclase in GI smooth muscle cells is dispensable for motility. Lack of NO-GC in both SMC and ICC, though, totally abolishes nitrergic signaling. However, in these dbl-GCKO mice strong NO-GC expression was still detected in FLC in gastrointestinal tissue. Further investigation is needed to elucidate the role of NO-GC in this cell type.

